# Auditory naming is impaired in posterior cortical atrophy and early-onset Alzheimer’s disease

**DOI:** 10.3389/fnins.2024.1342928

**Published:** 2024-01-24

**Authors:** Deepti Putcha, Ana Eustace, Nicole Carvalho, Bonnie Wong, Megan Quimby, Bradford C. Dickerson

**Affiliations:** ^1^Frontotemporal Disorders Unit and Alzheimer’s Disease Research Center, Departments of Psychiatry and Neurology, Massachusetts General Hospital, Boston, MA, United States; ^2^Harvard Medical School, Boston, MA, United States

**Keywords:** atypical AD, logopenic variant primary progressive aphasia, semantic network, confrontation naming, neuropsychology

## Abstract

**Introduction:**

Visual naming ability reflects semantic memory retrieval and is a hallmark deficit of Alzheimer’s disease (AD). Naming impairment is most prominently observed in the late-onset amnestic and logopenic variant Primary Progressive Aphasia (lvPPA) syndromes. However, little is known about how other patients across the atypical AD syndromic spectrum perform on tests of auditory naming, particularly those with primary visuospatial deficits (Posterior Cortical Atrophy; PCA) and early onset (EOAD) syndromes. Auditory naming tests may be of particular relevance to more accurately measuring anomia in PCA syndrome and in others with visual perceptual deficits.

**Methods:**

Forty-six patients with biomarker-confirmed AD (16 PCA, 12 lvPPA, 18 multi-domain EOAD), at the stage of mild cognitive impairment or mild dementia, were administered the Auditory Naming Test (ANT). Performance differences between groups were evaluated using one-way ANOVA and *post-hoc t*-tests. Correlation analyses were used to examine ANT performance in relation to measures of working memory and word retrieval to elucidate cognitive mechanisms underlying word retrieval deficits. Whole-cortex general linear models were generated to determine the relationship between ANT performance and cortical atrophy.

**Results:**

Based on published cutoffs, out of a total possible score of 50 on the ANT, 56% of PCA patients (mean score = 45.3), 83% of EOAD patients (mean = 39.2), and 83% of lvPPA patients (mean = 29.8) were impaired. Total uncued ANT performance differed across groups, with lvPPA performing most poorly, followed by EOAD, and then PCA. ANT performance was still impaired in lvPPA and EOAD after cuing, while performance in PCA patients improved to the normal range with phonemic cues. ANT performance was also directly correlated with measures of verbal fluency and working memory, and was associated with cortical atrophy in a circumscribed semantic language network.

**Discussion:**

Auditory confrontation naming is impaired across the syndromic spectrum of AD including in PCA and EOAD, and is likely related to auditory-verbal working memory and verbal fluency which represent the nexus of language and executive functions. The left-lateralized semantic language network was implicated in ANT performance. Auditory naming, in the absence of a visual perceptual demand, may be particularly sensitive to measuring naming deficits in PCA.

## 1 Introduction

Word retrieval difficulty is increasingly considered a core symptom of the typical late-onset amnestic syndrome of Alzheimer’s disease (AD), and is characterized by semantic verbal fluency and naming deficits (Salmon et al., 1999; Henry et al., 2004; Papp et al., 2016; Putcha et al., 2020). Since AD has been conceptualized biologically as a neuropathologic process leading to a syndromic spectrum, there has been a greater focus on characterizing the non-amnestic, atypical syndromes of AD including posterior cortical atrophy (PCA), logopenic variant of primary progressive aphasia (lvPPA), and single- and multi-domain syndromes of early onset AD (EOAD). While the language profile of lvPPA has been well-characterized as having primary difficulty with word retrieval, naming, sentence repetition, and phonological speech errors (Gorno-Tempini et al., 2008, 2011), we do not yet have a comprehensive understanding of how word retrieval difficulty may present in PCA and EOAD.

Early onset is defined as having symptom onset younger than age 65, but this syndrome is not simply the same as typical late-onset amnestic AD occurring at a younger age. The unique clinical characteristics of the heterogeneous EOAD syndrome are characterized by a more aggressive disease course with a greater severity of cognitive impairment in non-memory domains, including executive dysfunction and language (Palasi et al., 2015; Wattmo and Wallin, 2017; Hammers et al., 2023). It is important to note that EOAD studies by and large are comprised mainly of patients with either a dysexecutive or amnestic predominant presentation and do not typically include many PCA or lvPPA patients (e.g., Hammers et al., 2023) though the latter two syndromic categories can also present at a younger age of onset. In this study, we use the term “EOAD” to indicate that these are individuals who do not meet diagnostic criteria for PCA or lvPPA. Compared to late-onset AD, individuals with EOAD are thought to have greater difficulty with confrontation naming (Sa et al., 2012; Mendez, 2019). However, given that visual naming is a multimodal process including visual processing, object recognition, semantic processing, and goal-directed retrieval skills (Gleichgerrcht et al., 2015), the mechanisms underlying this phenomenon may be different than what is understood in typical amnestic AD. Similarly, little is known about anomia in PCA, a clinical syndrome primarily characterized by a progressive decline in higher-order visual cognitive skills and other posterior cortical functions (Benson et al., 1988; Tang-Wai et al., 2004; Crutch et al., 2017). In addition to these visual cognitive deficits, anomia and verbal fluency have also been identified in PCA (Crutch et al., 2013; Magnin et al., 2013; Putcha et al., 2018, 2020). Since naming ability has most frequently been evaluated neuropsychologically with visual confrontation naming tests (e.g., the Boston Naming Test), one difficulty with interpreting naming impairment in PCA has been disambiguating naming deficits from visual perceptual difficulty—a core feature of PCA. Thus, further investigation of word retrieval and naming difficulty in the absence of a visual cue is warranted.

The Boston Naming Test (BNT) is one of the most common assessments of naming ability in neuropsychological evaluation of dementia (Hobson et al., 2011). However, there are several limitations of this test in assessing anomia in the atypical AD syndromic spectrum. In addition to heavy visual processing and object recognition demands, which are major confounders for individuals with PCA and those with significant visuospatial impairment, the BNT may have a high false negative rate. One study indicated normal performance in as high as 59% of patients with very mild or mild AD (Domoto-Reilly et al., 2012). Thus, the BNT is not likely to be sensitive to detecting early stages of mild cognitive impairment which is an important aspect of cognitive assessment toward the goal of early diagnosis. An alternative, the Auditory Naming Test [ANT; (Hamberger and Seidel, 2003)] was developed to address these limitations by requiring participants to name an item that is described verbally. The process of auditory naming relies on auditory processing of the prompt, the retrieval and production of words, as well as semantic knowledge and having the vocabulary to name the described items (Hamberger et al., 2022). The ANT has been shown to have greater ecological validity than the BNT in correlating with subjective complaints of word retrieval difficulty; this was postulated to be due to the inclusion of more commonly used vocabulary (Hamberger and Seidel, 2003). Furthermore, the ANT also includes a measurement of the tip-of-the-tongue phenomenon, capturing response latency that may be more sensitive to subtle word retrieval impairment than a dichotomous correct/incorrect score. The ANT (but not the BNT) has been reported to be more specific in activating a circumscribed left-hemisphere language network, and when compared directly to the BNT, the ANT was found to be more sensitive to detecting naming difficulties in patients with AD and vascular dementia (Hirsch et al., 2016). Thus, the ANT is an excellent candidate to evaluate the presence of anomia across the atypical syndromic spectrum of AD, with particular usefulness in the PCA and EOAD syndromes.

The primary goal of the present study was to determine how individuals with each of the atypical AD syndromes—PCA, lvPPA, and EOAD—perform on the ANT. We analyzed immediate responses separately from responses given extra time (tip-of-the-tongue) and responses aided by phonemic cuing. Building on converging evidence of word retrieval deficits in these atypical AD syndromes, we hypothesized an impairment in immediate auditory naming performance. We also expected that all groups would benefit from extra time as well as phonemic cuing, though benefit from these aids would be limited in the lvPPA syndrome who, by definition, would demonstrate the greatest naming deficits. A secondary goal was to examine the neuropsychological correlates of ANT performance to understand the cognitive mechanisms underlying naming performance. Given that one needs to be able to process and maintain the verbal prompt and then quickly retrieve the name of the described item, we hypothesized that auditory-verbal working memory (digit span) and speeded word retrieval (verbal fluency) would be closely related to auditory naming deficits. Lastly, we sought to determine the neuroanatomical underpinnings of performance on the ANT, using measures of cortical atrophy across these atypical AD syndromes. We hypothesized that performance on the ANT would be related to atrophy in a focal pattern within the left-lateralized language network in regions associated with expressive language and semantic retrieval (left hemisphere inferior frontal and anterior temporal cortex) and would not be related to posterior temporal and occipitoparietal regions commonly associated with BNT performance.

## 2 Materials and methods

### 2.1 Participant characteristics

Data for this study were obtained from forty-six participants (16 PCA, 12 lvPPA, 18 EOAD; Table 1) in the Massachusetts General Hospital Frontotemporal Disorders Unit longitudinal cohort, including the Primary Progressive Aphasia program (Sapolsky et al., 2014) and Posterior Cortical Atrophy program (Putcha et al., 2018), which are affiliated with the Massachusetts Alzheimer’s Disease Research Center. All participants received a standard clinical evaluation comprising a comprehensive neurological and psychiatric history and exam and structured informant interviews following the Clinical Dementia Rating (CDR) protocol, and a separate neuropsychological battery including the National Alzheimer’s Coordinating Center (NACC) Uniform Data Set (UDS) version 2.0 or 3.0 battery. For each patient, clinical diagnostic formulation was performed through consensus conference, with each patient being classified based on all clinical information as having mild cognitive impairment or dementia (global clinical status), and then each patient’s cognitive-behavioral syndrome being diagnosed according to standard diagnostic criteria (Dickerson et al., 2017; Wong et al., 2019). Twenty-five patients met diagnostic criteria for PCA (Tang-Wai et al., 2004; Crutch et al., 2017), 23 patients met criteria for lvPPA (Gorno-Tempini et al., 2011), and 18 patients met criteria for EOAD with single- or multi-domain amnestic or non-amnestic syndromes (Ossenkoppele et al., 2015; Townley et al., 2020). Though having early onset of symptoms and having a PCA or PPA clinical syndrome are not mutually exclusive, in this study we have made them distinct. That is, if an individual patient had and early onset of symptoms (younger than age 65), and also met criteria for PCA or PPA, they were thus classified into those syndromic categories. If they had early onset of symptoms and did not meet criteria for PCA or PPA, they were categorized simply as “EOAD,” indicating likely multidomain impairment. The patient sample was further restricted to those participants who had imaging or cerebrospinal fluid (CSF) biomarker status consistent with AD. Some patients underwent neuroimaging sessions involving structural MRI, flortaucipir (FTP) PET, and amyloid (PiB or FBB) PET scans. Aβ positivity was determined by a combination of visual read and mean amyloid PET signal extracted from a cortical composite region of interest according to previously published procedures (Rabinovici et al., 2010; Villeneuve et al., 2015; Cho et al., 2023). Determination of tau and neurodegeneration positivity was conducted by visual read using internal methods similar to published work (e.g., Rabinovici et al., 2011; Fleisher et al., 2020; Sonni et al., 2020). Other patients underwent CSF sampling with results indicating abnormally low levels of CSF amyloid-β and abnormally high levels of CSF total tau and hyperphosphorylated tau (Shaw et al., 2009). This resulted in a final patient sample size of 16 A + T + N + PCA, 12 A + T + N + lvPPA participants, and 18 A + T + N + EOAD. Individuals were not recruited into this study if they had a primary psychiatric or other neurologic disorder including major cerebrovascular infarct or stroke, seizure, brain tumor, hydrocephalus, multiple sclerosis, HIV-associated cognitive impairment, or acute encephalopathy. This work was carried out in accordance with The Code of Ethics of the World Medical Association (Declaration of Helsinki) for experiments involving humans. All participants and their informants/caregivers provided informed consent in accordance with the protocol approved by the Mass General Brigham Human Research Committee Institutional Review Board in Boston, Massachusetts.

**TABLE 1:** Clinical characteristics of the A + T + N + atypical AD sample.

Demographics	ALL (*N* = 46)	EOAD (*N* = 18)	PCA (*N* = 16)	lvPPA (*N* = 12)
Age (years)	66.0 ± 8.8*	60.1 ± 5.6	70.1 ± 8.6	69.7 ± 8.7
Sex (M/F)	18/28	7/11	6/10	5/7
Education (years)	16.5 ± 2.5	17.0 ± 2.2	17.0 ± 2.0	15.2 ± 3.0
Handedness (R/L/ambidextrous)	43/1/2	18/0	14/0/2	11/1
MoCA	15.3 ± 6.6	14.4 ± 6.0	16.4 ± 7.2	15.4 ± 6.8
CDR global	CDR 0 (*N* = 3)	CDR 0.5 (*N* = 9)	CDR 0.5 (*N* = 10)	CDR 0 (*N* = 3)
	CDR 0.5 (*N* = 24)	CDR 1 (*N* = 9)	CDR 1 (*N* = 5)	CDR 0.5 (*N* = 6)
	CDR 1 (*N* = 17)		CDR 2 (*N* = 1)	CDR 1 (*N* = 3)
	CDR 2 (*N* = 1)			

EOAD, early onset Alzheimer’s disease; PCA, posterior cortical atrophy; lvPPA logopenic variant of primary progressive aphasia. Means and Standard Deviations (SD) are reported for continuous variables. MoCA, Montreal cognitive assessment; CDR, clinical dementia rating scale.

*Indicates a significant difference in age between EOAD and both PCA and lvPPA samples.

### 2.2 Neuropsychological assessments and analysis

Auditory naming was assessed using the Auditory Naming Test (Hamberger and Seidel, 2003; Hirsch et al., 2016, 2021). The ANT is a 50-item test which provides oral descriptions to one word responses. The performance metrics of interest included: the total number of correct responses on immediate recall (IR), total correct given up to 20 s to respond, reflecting the “tip of the tongue” phenomenon (IR + TOT), and total correct with phonemic cues (IR + TOT + PC). The number of items correctly named were analyzed, as well as compared to cutoff scores (Hirsch et al., 2016) and normative age- and education- stratified data (Hirsch et al., 2021). Performance differences between AD syndromic groups were investigated using one-way analysis of variance, with *post-hoc* independent sample *t*-tests to verify between group differences. Statistical significance was set to a threshold of *p* < 0.05. Primary hypothesis-driven analyses were conducted with no corrections for multiple comparisons applied. Statistical analyses were conducted in IBM SPSS Version 29.0 (Armonk, NY, USA). In order to compare the ANT to other commonly used measures of word retrieval, we also administered select trials of the Controlled Oral Word Association Test (Spreen and Strauss, 1991), with the measures of interest being the total number of correct words produced in 1 min trials to the letter cues “F,” “A,” and “S,” and to the category cue of “Animals.”

Tests scores from the remainder of the neuropsychological battery are presented in Table 2 to describe the cognitive profile of the participants in the current study. This battery included Digit Span Forward and Backward (longest spans), Auditory Addition and Subtraction, the UDS3 Craft Story memory encoding and delayed recall task (Craft Story in UDS3), the Word Picture Matching test from the UDS3 FTLD Module, the California Verbal List Learning 2nd Edition Short Form (CVLT-II-SF) test, Spatial Span Forward and Backward (longest spans), the Birmingham Object Recognition Battery (BORB) Single and Overlapping Object Identification tests, and the Visual Object Spatial Perception (VOSP) Number Location test. To determine the mechanistic specificity of contributions to word retrieval performance observed on the ANT, we conducted bivariate correlations with digit span and verbal fluency, as well as the VOSP number location and Spatial Span Backward, with the *a priori* hypotheses that ANT performance would be related to performance on digit span and verbal fluency, and not to the latter two tests of visuospatial cognition. In order to establish that word retrieval difficulty observed on the ANT was not merely reflective of semantic knowledge loss, we also examined performance on a test of semantic association, as well as the relationship between the Word Picture Matching test and ANT performance.

**TABLE 2:** Neuropsychological test data.

Neuropsychological assessment	EOAD (*N* = 18)	PCA (*N* = 16)	lvPPA (*N* = 12)
**Executive functions**			
Longest digit span forward^†^	4.8 ± 1.2	6.3 ± 1.9	4.2 ± 1.7
Longest digit span backward	2.8 ± 1.4	3.4 ± 1.6	2.7 ± 1.3
Auditory addition (/12)	8.1 ± 3.3	8.4 ± 3.4	8.8 ± 2.8
Auditory subtraction (/12)	6.2 ± 3.3	6.8 ± 3.2	6.1 ± 3.3
**Language**			
MINT correct (/32)	25.6 ± 5.3	23.7 ± 7.6	19.4 ± 8.1
MINT correct with phonemic cues (/32)	27.4 ± 3.8	24.0 ± 7.2	23.2 ± 5.4
ANT correct (/50)*^‡^	39.2 ± 9.8	45.3 ± 4.8	29.8 ± 11.9
ANT with phonemic cues (/50)*^‡^	44.1 ± 6.9	48.3 ± 2.9	36.7 ± 8.9
Letter fluency (FAS)^†^^‡^	18.9 ± 10.0	41.7 ± 15.3	17.0 ± 12.8
Category fluency (animals)	9.7 ± 5.0	11.9 ± 6.5	10.0 ± 4.7
Word-picture matching (/20)	19.7 ± 0.6	18.3 ± 4.2	19.3 ± 1.2
**Memory**			
Craft story immediate recall (/44)^†^^‡^	6.9 ± 4.3	12.1 ± 6.3	6.5 ± 4.8
Craft story delayed recall (/44)^†^	2.4 ± 2.6	9.2 ± 5.9	5.4 ± 4.3
CVLT-II-SF total recall (/36)	14.7 ± 5.9	19.5 ± 6.9	14.0 ± 6.3
CVLT-II-SF SDFR (/9)	2.9 ± 1.8	4.1 ± 2.5	3.4 ± 2.2
CVLT-II-SF LDFR (/9)	1.6 ± 1.9	3.1 ± 3.3	3.3 ± 2.9
CVLT-II-SF recognition hits (/9)	7.7 ± 0.9	6.9 ± 2.5	7.6 ± 2.0
**Visuospatial**			
Longest spatial span forward^†^^‡^	3.4 ± 0.8	2.0 ± 1.5	3.9 ± 1.0
Longest spatial span backward^*^^‡^	1.8 ± 1.6	2.0 ± 1.5	3.3 ± 1.5
BORB single object identification (/40)^†^^‡^	38.3 ± 2.8	33.2 ± 5.0	38.5 ± 3.0
BORB overlapping object identification (/40)^†^^‡^	33.5 ± 7.3	18.3 ± 6.0	38.0 ± 3.4
VOSP number location test (/10)^‡^	5.5 ± 3.2	3.7 ± 2.8	7.2 ± 2.3

EOAD, early onset Alzheimer’s disease; PCA, posterior cortical atrophy; lvPPA, logopenic variant of primary progressive aphasia; Means and Standard Deviations (SD) are reported; MINT, multilingual naming test from the uniform data set; ANT, auditory naming test; CVLT-II-SF, California verbal learning test- 2nd edition—Short form; SDFR, short delay free recall; LDFR, long delay free recall; LDCR, long delay cued recall; BORB, Birmingham object recognition battery; VOSP, visual object spatial perception test.

*Indicates statistical significance between scores of EOAD and lvPPA.

^†^Indicates statistical significance between scores of EOAD and PCA.

^‡^indicates statistical significance between scores of lvPPA and PCA, all at the threshold of *p* < 0.05.

### 2.3 Neuroimaging acquisition and analysis

All participants in the final sample received a structural 3D T1-weighted scan at MGH. Scans were acquired using a Siemens Trio 3T scanner (Siemens Medical Systems). T1 image volumes were examined qualitatively by a cortical surface-based reconstruction and analysis of cortical thickness using FreeSurfer version 6.0.^1^ The general procedures for this processing method have been described in detail and applied and validated in a number of publications and presentations; the technical details can be found in select manuscripts (Dale et al., 1999; Fischl and Dale, 2000; Fischl et al., 2002, 2004).

All but 3 participants (1 PCA and 2 lvPPA) underwent ^11^C- Pittsburgh Compound B (amyloid) and FTP PET scans, which were spherically registered to align each individual’s cortical surface between PET and MR scans. The ^11^C-PiB PET scans were acquired with an 8.5 to 15 mCi bolus injection followed immediately by a 60-min dynamic acquisition in 69 frames (12 × 15 s, 57 × 60 s). FTP PET scans were acquired 80 to 100 min after the bolus injection of ∼10.0 mCi of FTP (4 × 5 min frames). All PET data were acquired using a Siemens/CTI (Knoxville, TN) ECAT HR + scanner (3D mode; 63 image planes; 15.2 cm axial field of view; 5.6 mm transaxial resolution and 2.4 mm slice interval). Data were reconstructed and attenuation corrected; each frame was evaluated to verify adequate count statistics; interframe head motion was corrected prior to further processing. Visual inspection confirmed accurate registration between anatomical and PET volumes. To evaluate the anatomy of PET binding, each individual’s PET data set was rigidly co-registered to the subject’s MPRAGE data using SPM8 (Wellcome Department of Cognitive Neurology, Function Imaging Laboratory, London). Similar to a previous report, ^11^C-PiB PET data were expressed as the distribution volume ratio (DVR) with the cerebellar gray matter as a reference (Becker et al., 2011), where regional time-activity curves (TAC) were used to compute regional DVRs for each ROI using the Logan graphical method applied to data from 40 to 60 min after injection. FTP standard uptake value ratio (SUVR) images were derived with whole cerebellar gray matter as a reference region. PET data were not partial volume corrected and were performed using geometric transform matrix as implemented in FreeSurfer stable release version 6.0. The remaining three participants were deemed to be A + T + N + positive based on clinically obtained CSF samples as described above.

To determine if performance on the ANT was related to cortical atrophy in our hypothesized regions, we conducted whole cortical surface general linear models (GLM) for the effects of the task performance on cortical thickness at each vertex point on the cortical surface. Using methods we have previously published (Dickerson et al., 2008; Makaretz et al., 2017; Xia et al., 2017; Putcha et al., 2020), we used age-, education-, and sex- adjusted performance scores and thus did not control for these demographic factors again in our cortical thickness GLM analysis. Follow-up analysis ensured that cortical thickness was not related in any significant way to any of these demographic factors. GLM analyses was implemented using the *mri_glmfit* utility within FreeSurfer version 6. Given our specific *a priori* hypotheses, an uncorrected statistical threshold of *p* < 0.001 was set.

## 3 Results

### 3.1 Clinical characteristics

A total of 46 A + T + N + symptomatic individuals across the atypical syndromic spectrum of AD (16 PCA, 12 lvPPA, 18 EOAD) were included in this study. See Table 1 for complete sample characteristics. Consistent with the diagnostic criteria of “early onset” AD, we observed that our EOAD group were significantly younger than our PCA (*t* = 4.1, *p* = 0.0001) and lvPPA (*t* = 3.7, *p* = 0.001) groups. The average age of PCA and lvPPA individuals were comparable. There were no syndromic group differences in years of education or sex. All but one patient included in these analyses were rated at the stage of mild cognitive impairment or mild dementia. Some individuals were mildly symptomatic in their primary domains of impairment (i.e., language) though this was not captured on the traditional CDR scale thus giving them a CDR of 0, although their CDR supplemental language box score was 0.5 or 1. Participants from all groups were at the relatively same stage of cognitive decline; the mean MoCA scores for the whole sample was 15.3 out of 30, with no statistically significant difference among the three syndromic groups (*p* > 0.7). All individuals underwent comprehensive neuropsychological testing, including assessments of Attention and Executive Functioning, Language, Memory, and Visuospatial Skills. See Table 2 for a full neuropsychological characterization.

### 3.2 Auditory naming is impaired across the atypical AD syndromes (lvPPA, EOAD, PCA)

**FIGURE 1:**
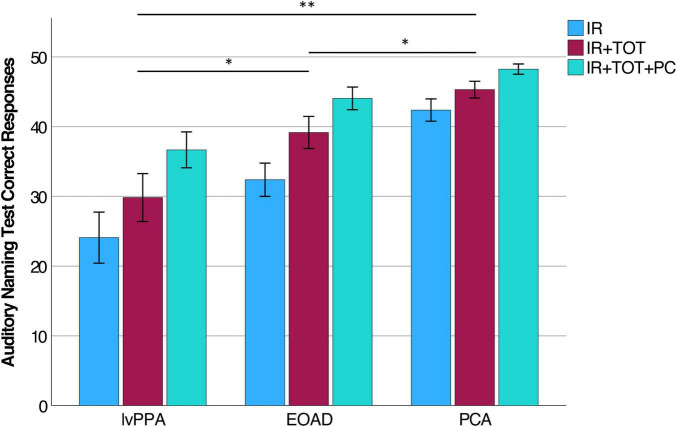
Auditory Naming Test performance in lvPPA, EOAD, and PCA. Within each diagnostic group, participants performed most poorly on IR and experienced incremental benefit to producing correct responses given extra time (IR + TOT) and after given a phonemic cue (IR + TOT + PC). Each group was statistically different from each other on performance on the IR + TOT variable specifically (lvPPA < EOAD < PCA). *Indicates *p* < 0.05, **Indicates *p* < 0.005.

**FIGURE 2:**
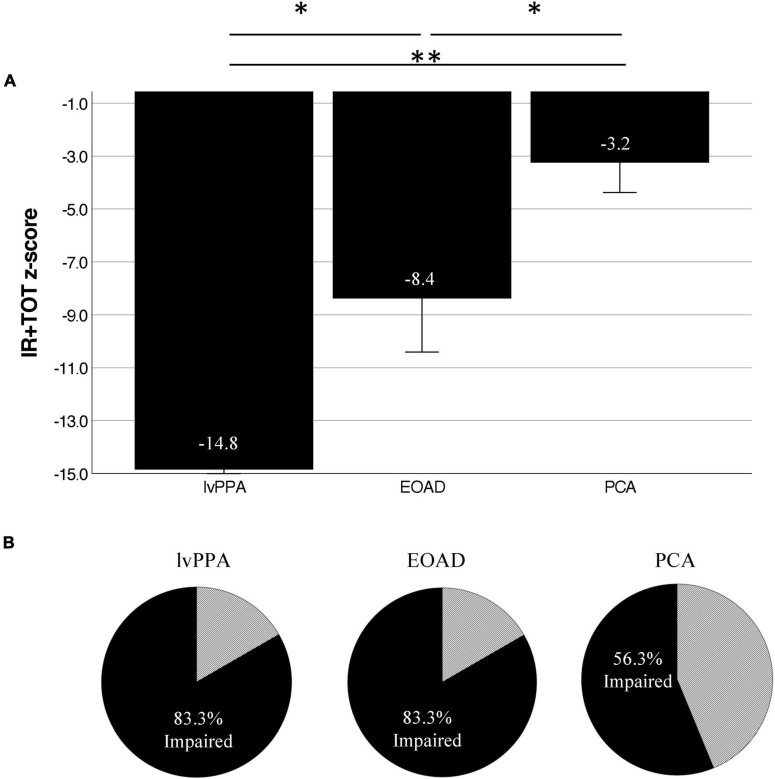
Atypical AD patients are normatively impaired on the Auditory Naming Test. **(A)** Normalized performance (z-scores) of ANT IR + TOT performance in patients compared to age- and education-matched control participants demonstrate severe impairment across groups on correct uncued responses. **(B)** Using Hirsch et al. (2016) impairment cutoffs, we demonstrate that 83.3% of lvPPA and EOAD patients, as well as 56.3% of PCA patients are below the cutoff for impairment. *Indicates *p* < 0.05, **Indicates *p* < 0.005.

We measured three variables of the Auditory Naming Test: the number of correct items correctly named within 2 s (immediate recall or “IR”), the number of additional items correctly named given extra time (tip-of-the-tongue or “TOT”), and the number of items correctly named with a phonemic cue (“PC”). Comparing the IR to the IR + TOT scores, we observed that all groups benefitted from extra time. All benefitted further from phonemic cuing. On all three variables, the lvPPA group performed most poorly followed by the EOAD group and finally the PCA group (see Table 3). We focused the remainder of our analyses on the IR + TOT score, as previous publications (Hirsch et al., 2016) have identified this variable as most sensitive to measuring naming impairment in the dementia population. We found significant differences between groups on IR + TOT performance, such that lvPPA performed worse than EOAD (*t* = 1.9, *p* = 0.03) and worse than the PCA group (*t* = 4.7, *p* < 0.001). The EOAD group also performed worse than the PCA group (*t* = 2.3, *p* = 0.04; Figure 1). Notably, all groups performed in the normatively impaired range even with the extra time (Figure 2A), with z-scores falling below the 1st percentile (*z* < −3.0). Using the age- and education- stratified impairment cutoffs published by Hirsch et al. (2016), we discovered that 83.3% of individuals with lvPPA and EOAD, respectively, and more than half (56.3%) of individuals with PCA are considered impaired (Figure 2B). Finally, all participant groups benefitted from phonemic cuing, with PCA patients performing within normal limits on this fully cued condition of the ANT.

**TABLE 3:** Auditory naming test.

Neuropsychological assessment	EOAD (*N* = 18)	PCA (*N* = 16)	lvPPA (*N* = 12)
ANT IR^‡^^†^	32.4 ± 10.1	42.4 ± 6.4	24.1 ± 12.7
ANT TOT^†^	6.8 ± 5.2	2.9 ± 3.7	5.8 ± 4.3
ANT IR + TOT (/50)*^‡^^†^	39.2 ± 9.8	45.3 ± 4.8	29.8 ± 11.9
ANT IR + TOT z-score^‡^^†^	−8.4 ± 8.6	−3.2 ± 4.5	−14.8 ± 9.6
ANT IR + TOT + PC (/50)*^‡^^†^	44.1 ± 6.9	48.3 ± 2.9	36.7 ± 8.9

ANT subscores. IR = Immediate Responses within 2 s. TOT = Tip-of-tongue responses given between 2 and 20 s. PC = Phonemic Cuing. IR + TOT represents the total number of correct responses within 20 s. IR + TOT + PC represents the total number of correct responses after a phonemic cue is given.

*Indicates statistical significance between scores of EOAD and lvPPA.

^†^Indicates statistical significance between scores of EOAD and PCA.

^‡^Indicates statistical significance between scores of lvPPA and PCA, all at the threshold of *p* < 0.05.

### 3.3 Auditory naming depends on working memory and word retrieval

**FIGURE 3:**
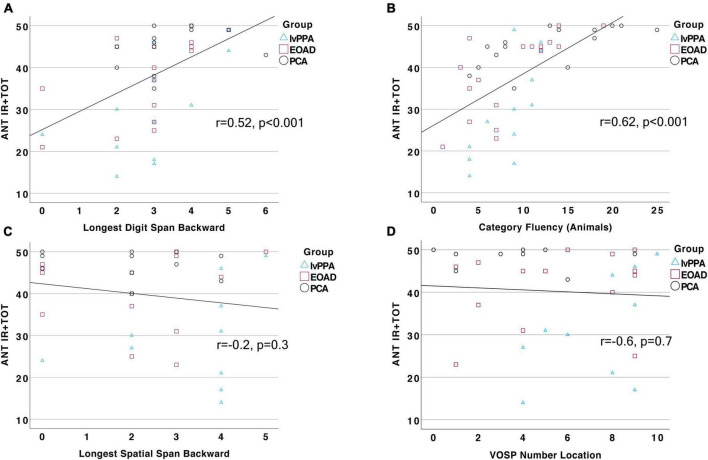
Auditory naming performance is correlated with measures of working memory and verbal fluency. Total correct response (IR + TOT) on the ANT is strongly and positively correlated to performance on **(A)** Longest Digit Span Backward (*r* = 0.52, *p* = 0.0003) and **(B)** Category Fluency (*r* = 0.62, *p* = 0.000006), but not related to performance on **(C)** Longest Spatial Span Backward, *p* > 0.1, or **(D)** VOSP Number Location test, *p* > 0.1.

To better understand the cognitive processes contributing to the ability to perform an auditory naming task, we tested our *a priori* hypotheses that auditory naming would be related to auditory-verbal working memory as well as goal-directed speeded lexical retrieval. We identified the Digit Span Backward test as a reliable measure of working memory and tests of verbal fluency—to both letter and category cues—as measures of speeded lexical retrieval. Across our whole sample, we found that ANT IR + TOT performance was strongly correlated with Longest Digit Span Backward (*r* = 0.52, *p* = 0.0003; Figure 3A), letter fluency (FAS; *r* = 0.48, *p* = 0.006), and category fluency (Animals; *r* = 0.62, *p* = 0.000006; Figure 3B). Of note, letter and category fluency performance was highly correlated (*r* = 0.65, *p* = 0.0001), and letter fluency did not explain unique variance over and above category fluency in a multiple regression model (*p* > 0.1). We also found that ANT IR + TOT performance was correlated to a global screening measure of cognition (MOCA; *r* = 0.58, *p* = 0.00002). We then assessed the relationship between ANT performance and tests of visuospatial cognition, which should not be correlated, to determine the specificity of relationships to our hypothesized cognitive constructs. We chose two visuospatial tests from our battery, the VOSP Number Location Test and Longest Spatial Span Backward, to represent visual attention and space perception. We found that ANT IR + TOT performance was not correlated with either the Longest Spatial Span Backward (*r* = −0.2, *p* = 0.3; Figure 3C) nor the VOSP Number Location Test (*r* = −0.6, *p* = 0.7; Figure 3D). Lastly, we examined the association between ANT performance and a non-verbally mediated test of semantic knowledge, Word Picture Matching. All participant groups performed well on this test (Table 2) indicating intact semantic knowledge. Across the whole group, we did not observe a significant relationship between performance on the Word Picture Matching test and ANT IR + TOT (*r* = 0.4, *p* = 0.3), suggesting that the semantic word retrieval impairment we observe on the ANT is not explained by any loss of the actual semantic knowledge regarding the items being tested. These associations did not change in strength when demographic and clinical factors (e.g., disease severity) were controlled for.

### 3.4 Auditory naming performance is related to atrophy in a semantic retrieval network

**FIGURE 4:**
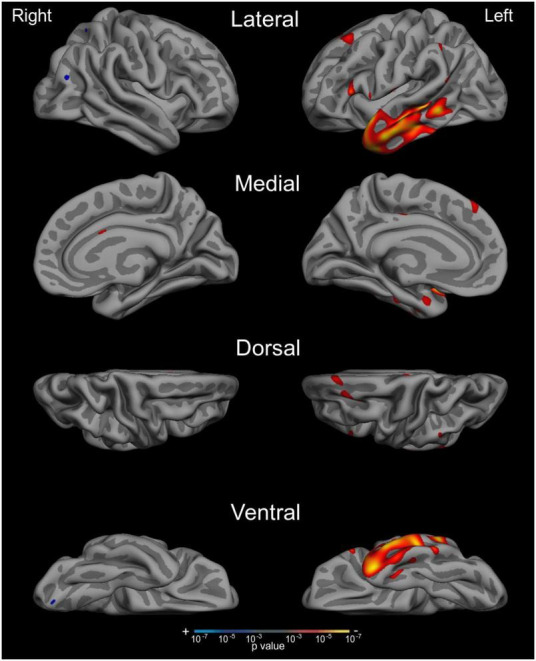
Auditory naming impairment is associated with atrophy in the left anterior lateral temporal cortex across the AD syndromic spectrum. Whole cortex general linear models demonstrate that reduced cortical thickness in anterior and middle temporal regions was associated with worse performance on the ANT IR +TOT measure. Results show maps of *p*-values, thresholded at *p* < 0.001.

We next tested our *a priori* hypotheses regarding the neuroanatomical correlates of auditory naming by conducting a whole-cortex general linear model predicting ANT IR + TOT performance. We combined our whole sample together for these analyses in an effort to capitalize on the heterogeneity in cognitive profile and neurodegeneration across syndromic groups. We found that ANT performance was correlated with circumscribed atrophy in the left anterior lateral temporal lobe extending posteriorly into the middle temporal gyri, as well as in the inferior frontal gyrus (Figure 4; *p* < 0.001). We did not observe any atrophy in the visual association cortices or frontoparietal networks as has been observed with traditional tests of visual confrontation naming.

## 4 Discussion

Word-retrieval and naming difficulties are consistently reported areas of impairment in AD, particularly in the typical older-onset amnestic syndrome and the logopenic variant of Primary Progressive Aphasia (Salmon et al., 1999; Henry et al., 2004; Papp et al., 2016; Leyton et al., 2017; Putcha et al., 2020). While some evidence of naming deficits have emerged in other atypical variants of AD, including PCA (Crutch et al., 2013; Magnin et al., 2013; Putcha et al., 2020) and EOAD (Sa et al., 2012; Mendez, 2019), the majority of methods used to assess naming in these populations have been visual confrontation naming tests such as the BNT or Neuropsychological Assessment Battery Naming subtest. The heavy visual perceptual demand inherent in these tests presents a major confound, and thus makes them unsuitable evaluation tools to measure anomia in PCA and other clinical syndromes with visuospatial or perceptual cognitive impairment. By analyzing naming performance on the ANT, we found that the lvPPA group was the most severely impaired compared to our EOAD and PCA, as expected. Critically, we also found that the PCA and EOAD participants demonstrated significant anomia. Over 83% of EOAD participants and over 56% of PCA participants were found to be impaired on this test based on previously published impairment cut-off scores (Hirsch et al., 2016). We thus add converging knowledge to the literature that EOAD is characterized by naming impairment, whether the stimuli are visual or auditory in nature. Moreover, we contribute more convincingly to the emerging literature describing a “logopenia” syndrome in PCA (Crutch et al., 2013; Putcha et al., 2018, 2020; Tetzloff et al., 2021) by demonstrating that a significant proportion of individuals with PCA, at early stages of disease progression, have naming difficulty even on tests that do not require perceptual processing of a visual stimulus. Despite the diagnostic consensus criteria of PCA specifying a relative sparing of domains including language, memory, and executive functions (Crutch et al., 2017), our field continues to have a growing understanding of how early in the disease process these “secondary domains of impairment” can be impacted. We hope these results will inform the use of auditory naming tests in routine neuropsychological evaluation in PCA.

A secondary goal of this study was to investigate the cognitive contributors to auditory naming performance across the atypical AD syndromic spectrum. We found that ANT performance was strongly associated with performance on tests of auditory-verbal working memory (digit span backward) and category fluency. These are tests on which we and others have demonstrated mild impairment in PCA at the stage of MCI consistent with the neurodegeneration observed in posterior temporoparietal cortices supporting working memory and lexical retrieval abilities. We have previously demonstrated that deficits in working memory and verbal fluency are related to memory encoding and retrieval, and we here add to that formulation by suggesting that these areas of weakness also impact auditory name retrieval. We did not observe any associations between ANT performance and tests of visual cognition, highlighting the specificity of associations to our hypothesized domains of interest. We conclude that auditory naming ability may depend on auditory-verbal working memory and goal-directed lexical retrieval in this population. Indeed, all participants benefitted from receiving phonemic cuing, indicating that the primary deficit is one of word retrieval rather than semantic memory loss.

Lastly, we sought to investigate the anatomical underpinnings of ANT performance in this atypical AD sample, capitalizing on the heterogeneity of neurodegenerative profiles across these syndromic variants. Whole-cortex general linear modeling examining the relationship between ANT performance and cortical atrophy revealed an association specifically in the left hemisphere anterior temporal cortex extending posteriorly within the middle temporal gyrus (MTG). These observations converge with what is known about the brain structures supporting semantic memory processes and object naming (Sapolsky et al., 2010; Rogalski et al., 2011; Ralph et al., 2017). Our results also align with previous work reporting left-lateralized anterior temporal cortical atrophy in typical late-onset AD patients with impairment on a visual naming task, though in that study there was less middle temporal and greater ventral temporal involvement (Domoto-Reilly et al., 2012). In addition to the semantic “hub” that the anterior temporal lobes represent (Ralph et al., 2017), we observed correlations between auditory naming performance and atrophy in the left posterior MTG, which we have written about previously (Putcha et al., 2020) as representing part of the controlled lexical retrieval network supporting the integration of goal-directed retrieval and specific semantic information—i.e., the “lexical-semantic interface in Hickok and Poeppel’s model” (Hickok and Poeppel, 2007). Compared to our previous publication examining BNT performance in this same atypical AD population (Putcha et al., 2020), the atrophy pattern we see here in relation to ANT performance is much more circumscribed to semantic and lexical retrieval nodes of the language network compared to the more diffuse pattern of cortical atrophy observed in relation to BNT performance which included bilateral inferior temporal and visual association cortices. Taken together with previous work purporting that the ANT is more clinically sensitive to impairment at earlier stages of disease progression compared to the BNT (Hamberger and Seidel, 2003), these neuroanatomical relationships also indicate that this test may be a more “process pure” interrogation of auditory-verbal, semantic, and lexical retrieval abilities.

There are some limitations to this study that would be important to consider when interpreting these results. The first is that the stimuli included in the ANT are all concrete and “visualizable” words (e.g., “wrinkle”). That is, none of the items described are conceptual in nature, and thus may actually be more challenging for PCA participants than abstract word naming (e.g., “aging”). This is a topic that warrants further investigation to determine if a “pure” naming deficit is part of the PCA syndrome, or if the anomia we observe in this study is particular to the nature of stimuli presented, even in a naming task that is entirely verbally administered and answered. A second limitation is our relatively small sample size. Given that we are studying atypical and rare AD syndromes, our sample sizes in this study of each syndromic subgroup were small-to-moderate. Though on par with most studies of these diagnostic cohorts, it will be important to replicate these findings on a larger scale to ensure the robustness of our conclusions. Reassuringly, our results fall in line with our strong *a priori* hypotheses expecting severe anomia in lvPPA and EOAD, and an emerging logopenic syndrome in PCA. We hope that our observations reported here contribute to the characterization of atypical AD syndromes and will facilitate earlier diagnosis as well as monitoring of clinical symptoms in studies or management of the atypical syndromic spectrum of AD.

## Data availability statement

The raw data supporting the conclusions of this article will be made available by the authors, without undue reservation.

## Ethics statement

The studies involving humans were approved by the MassGeneral Brigham Human Research Committee Institutional Review Board. The studies were conducted in accordance with the local legislation and institutional requirements. The participants provided their written informed consent to participate in this study.

## Author contributions

DP: Conceptualization, Data curation, Formal analysis, Funding acquisition, Investigation, Methodology, Project administration, Writing – original draft, Writing – review and editing. AE: Data curation, Formal analysis, Investigation, Project administration, Writing – original draft. NC: Data curation, Formal analysis, Investigation, Visualization, Writing – original draft. BW: Conceptualization, Methodology, Writing – review and editing. MQ: Conceptualization, Data curation, Writing – review and editing. BD: Conceptualization, Funding acquisition, Methodology, Resources, Supervision, Writing – review and editing.

## Footnotes

1
http://surfer.nmr.mgh.harvard.edu
